# Digital Gamification to Enhance Vaccine Knowledge and Uptake: Scoping Review

**DOI:** 10.2196/16983

**Published:** 2020-05-18

**Authors:** Ilaria Montagni, Inass Mabchour, Christophe Tzourio

**Affiliations:** 1 Bordeaux Population Health U1219 Inserm-University of Bordeaux Bordeaux France; 2 Institute of Public Health Epidemiology and Development (ISPED) University of Bordeaux Bordeaux France; 3 Faculty of Medicine Hyacinthe Bastaraud University of Antilles-Guyane Pointe-à-Pitre Guadeloupe

**Keywords:** gamification, vaccination, vaccine hesitancy, digital tools, scoping review

## Abstract

**Background:**

Vaccine hesitancy is a growing threat to population health, and effective interventions are needed to reduce its frequency. Digital gamification is a promising new approach to tackle this public health issue.

**Objective:**

The purpose of this scoping review was to assess the amount and quality of outcomes in studies evaluating gamified digital tools created to increase vaccine knowledge and uptake.

**Methods:**

We searched for peer-reviewed articles published between July 2009 and August 2019 in PubMed, Google Scholar, Journal of Medical Internet Research, PsycINFO, PsycARTICLES, Psychology and Behavioral Sciences Collection, and SocINDEX. Studies were coded by author, year of publication, country, journal, research design, sample size and characteristics, type of vaccine, theory used, game content, game modality, gamification element(s), data analysis, type of outcomes, and mean quality score. Outcomes were synthesized through the textual narrative synthesis method.

**Results:**

A total of 7 articles met the inclusion criteria and were critically reviewed. Game modalities and gamification elements were diverse, but role play and a reward system were present in all studies. These articles included a mixture of randomized controlled trials, quasi-experimental studies, and studies comprising quantitative and qualitative measures. The majority of the studies were theory-driven. All the identified gamified digital tools were highly appreciated for their usability and were effective in increasing awareness of vaccine benefits and motivation for vaccine uptake.

**Conclusions:**

Despite the relative paucity of studies on this topic, this scoping review suggests that digital gamification has strong potential for increasing vaccination knowledge and, eventually, vaccination coverage.

## Introduction

Vaccination is one of the most cost-effective methods of preventing the spread of infectious diseases. The rates of people receiving vaccinations have recently declined in developed countries. If vaccination coverage falls below the thresholds that are safe for the prevention of epidemic transmission, the incidence of vaccine-preventable diseases increases [1]. One of the most illustrative examples of this phenomenon is the measles outbreak that returned over the past 2 decades. In the first 6 months of 2019, reported measles cases were the highest they had been in any year since 2006, indicating a concerning and continuing upsurge in the overall measles burden worldwide [2].

Reasons why some people do not get vaccinated are as varied as they are complex and include a sense of complacency, difficulty in accessing vaccines, mistrust of health or medical authorities, spread of misinformation, underestimation of risks, and limited knowledge of the benefits of vaccination and how it works [3,4]. According to a 2019 World Health Organization report, “vaccine hesitancy” (ie, the reluctance or refusal to be vaccinated) is one of the top 10 threats to global health [5]. Interventions to address vaccine hesitancy are urgently needed in order to promote vaccine acceptance and uptake in developed countries.

Many researchers have explored different ways to deliver fair information on vaccine risks and benefits through ad hoc interventions addressing different targets, ranging from parents [6] to adolescents [7], and concerning different types of vaccines, ranging from human papilloma virus (HPV) vaccination [8] to measles, mumps, and rubella vaccination [9]. In order to better design and implement such interventions, Willis et al [10] proposed a classification of 7 important items to be used in immunization communication methods: inform or educate, remind or recall, teach skills, provide support, facilitate decision making, enable communication, and enhance community ownership.

Gamification is defined as the use of game design elements in non-game contexts [11]. It encompasses several features and dimensions like fun interfaces, immediate success or continuous progress feedback, reward systems (point scores, badges, levels), challenges and competitions, team playing, avatars, and quizzes. These features echo the 7 items described by Willis et al [10] for efficacious immunization communication. Previous studies have been conducted worldwide on the use of gamification as a means to increase the initiation and retention of desired health behaviors [12-14]. By using game-based mechanisms, gamification stimulates participants’ involvement and facilitates their learning about health [15]. Serious games and mobile or tablet applications with game-based features are increasingly used to not only train health professionals but also deliver prevention and health promotion messages to the general population [16,17]. In detail, serious games are defined as full-blown digital games applied to train and educate players and are not predominantly or exclusively intended for entertainment purposes [18,19]. On the other hand, gamified digital tools (eg, apps) are not a full game experience but just contain gaming elements such as scoring of points, in-game rewards, or engaging in quests [20]. Thus, gamification is a broader concept including but not limited to serious games. A literature review of empirical studies on gamification [21] has provided evidence on the effectiveness of the game-based approach on the user, particularly on motivation and engagement.

Given its increasing use in the public health domain, gamification might be a useful approach for interventions aimed to sensitize populations to the relevance of vaccination acceptance. Including game-based features might improve vaccination campaign strategies by educating individuals, explaining the risks they face if they are not vaccinated and encouraging them to keep their vaccination records up to date. Interventions using fun and interactive approaches and leveraging digital technologies to deliver positive views on vaccination are increasingly requested [22]. A previous systematic review identified 16 serious games related to vaccination developed from 2003 to 2015 [23]. However, data on the effectiveness of these tools were not fully provided nor compared. Among the 16 serious games, only 2 games were formally evaluated [23,24]. Furthermore, other vaccination-related gamified digital tools, like mobile apps or quizzes, were not taken into account. A study on the evaluation of existing gamified digital tools for vaccination not limited to serious games would bridge this research gap.

The aim of this scoping review was to identify gamified digital tools that have been implemented and evaluated across diverse populations and types of vaccines in an effort to tackle issues of vaccine hesitancy. The effectiveness of identified tools in terms of impact on users’ knowledge and behavior towards vaccination as well as their usability/acceptability were also synthetized. Thus, the overarching goal of this study was to respond to the need for information on evidence-based interventions that could help design and implement future gamified digital tools to address vaccination hesitancy.

## Methods

### Search Strategy

We conducted a scoping review using the Preferred Reporting Items for Systematic Reviews and Meta-Analyses statement as a more robust methodological approach [25]. The search was performed between July 2019 and August 2019.

Before starting the review, we manually checked for relevant articles in the authors’ languages (ie, French, Italian, and Spanish) on the first 20 pages of Google Scholar. Since we did not find any article on “gamification” AND “vaccine” in corresponding languages, we confirmed our initial choice to restrict our search to English-language papers. The following search terms and related variations were used: all (“vaccine*” OR “vaccination” OR “immunization*”) AND all (“serious game*” OR “video game*” OR “therapeutic game*” OR “online game*” OR “game*” OR “game app*” OR “mobile game*” OR “digital game*” OR “gamif*”).

Studies were selected from a search of the following major electronic databases: PubMed, Google Scholar, Journal of Medical Internet Research, PsycINFO, PsycARTICLES, Psychology and Behavioral Sciences Collection, and SocINDEX via EBSCOhost. A supplementary manual search was performed to identify additional relevant publications by reviewing the reference lists of the included articles and using ResearchGate.

### Selection Criteria

Only peer-reviewed studies written in English in the decade 2009-2019 were included, regardless of the location of the study, type of vaccination under study, and study population. All types of study designs were included (eg, quantitative, qualitative, and mixed-methods studies; systematic reviews; meta-analyses; randomized controlled trials; and other experimental studies like pretests and posttests). Posters, preprints, and conference proceedings were excluded. Studies were included only if they used a gamification technique or tool to deliver informative or educative messages on vaccination. All types of digital games or gamified elements were included, from serious games (content gamification) to gamified Web-based quizzes (structural gamification) [26]. Articles not presenting the description and evaluation of a concrete gamified digital tool were excluded (eg, articles reporting the results of a survey on users’ needs and perspectives on games for vaccination).

### Data Extraction and Quality Assessment

Records identified in the literature search were evaluated in a 3-step approach. First, all identified titles and abstracts were screened for eligibility and coded by one researcher according to the selection criteria. Second, relevant articles were retrieved, and full-text articles were read independently by the researcher in charge of coding and extracting all data and by a second researcher. Third, a final list of publications for full-text review was established and validated by the 2 researchers. Any discrepancies were reviewed by a third researcher and finally resolved through consensus.

The 2 researchers conducted a quality assessment using the quality assessment method presented by Connolly et al [27]. This method assesses the overall weight of empirical evidence for the positive impact and outcomes of games. Each final paper included in the review was given a score of 1 (low), 2 (medium), or 3 (high) across the following 5 criteria: (1) appropriateness of the research design, with a score of 3 for randomized controlled trials, 2 for quasi-experimental controlled studies, and 1 for case studies, single subject-experimental, pretest/posttest, and other types of quantitative and qualitative studies; (2) appropriateness of methods and analysis; (3) representativeness and generalizability of the findings; (4) relevance of the focus of the study; and (5) relevance of the findings and their discussion. The total score for each paper was calculated by adding the scores of all 5 dimensions, resulting in a range from 5 to 15 points. Following the studies by Connolly et al [27] and Johnson et al [13] using the same quality assessment method, we categorized articles with a score of ≤8 points as weaker evidence, articles with a rating >8 to 12 points as moderate evidence, and articles with a rating >12 points as stronger evidence. We calculated the average score for each study and measured the weighted Cohen's kappa coefficient to test interrater reliability.

Data were sorted in categories, including author, year of publication, country, journal, research design, sample size and characteristics, type of vaccine, theory used, game content, game modality, gamification element(s), data analysis, type of outcomes, and mean quality score. For game modality and gamification elements, we based our coding on the work by Hamari et al [21].

### Outcome Measures

To assess the effectiveness of gamified digital tools for vaccination, we took into account 3 types of outcomes: behavior (eg, real actions like receiving a vaccination or intent to get vaccinated), cognition (eg, increased knowledge of the topic or vaccine literacy [28]), and usability/acceptability (eg, appreciation of the intervention). Furthermore, detailed outcomes of the evaluation of each game were individually reported.

## Results

**Figure 1:**
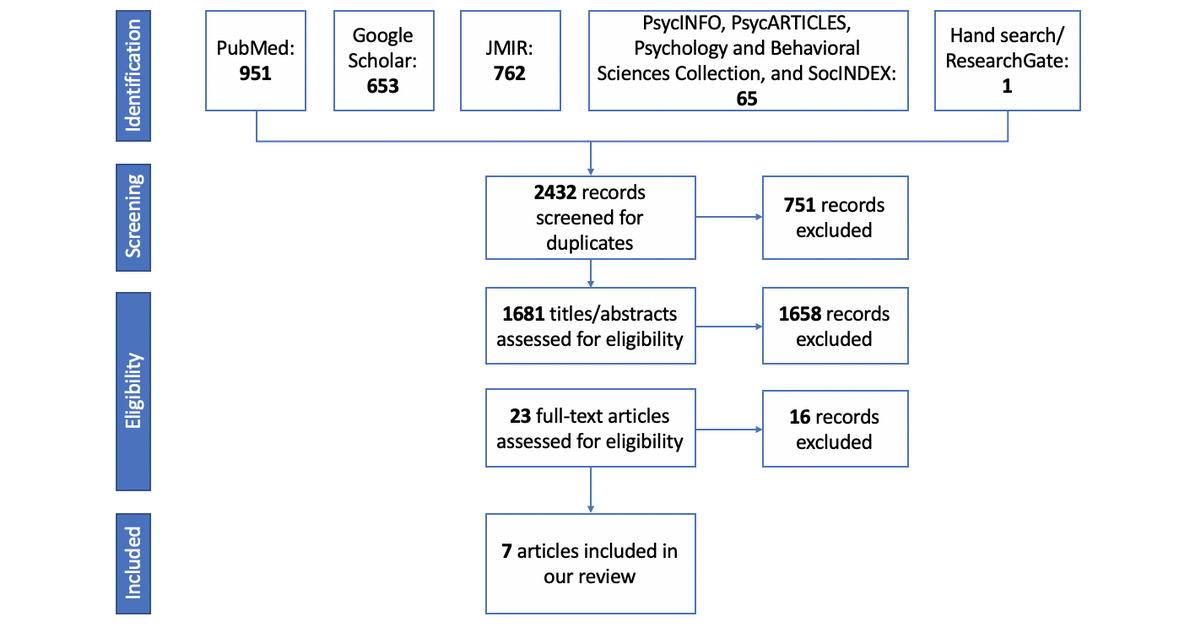
Flow diagram of the literature search according to the Preferred Reporting Items for Systematic Reviews and Meta-Analyses (PRISMA) statement.

A total of 2432 records were identified through database searches. After duplicates were removed, remaining papers were assessed using the described selection criteria. The same tool was presented by 2 different papers [29,30], with similar results. We decided to select only the most recent study [30] since it provided more in-depth information, including qualitative data. As a result, 7 articles were finally included. Figure 1 shows the flowchart of the study selection process.

### General Description of the Studies

Each study presented 1 gamified digital tool, with the exception of 1 study that described and evaluated 2 tools: one on vaccination and one on antibiotics [31]. Only data concerning the vaccination game were taken into account for this scoping review.

The final 7 articles eligible for review were rated for the quality of the evidence: 2 articles were categorized as providing weaker evidence [24,32], 2 articles were categorized as providing moderate evidence [33,34], and 3 articles were categorized as providing stronger evidence [29,31,35]. The weighted Cohen's kappa coefficient was 0.71, suggesting good agreement between raters [36]. Concerning methodologies, 2 studies used a randomized controlled trial design [29,34], 1 study used a controlled experiment with posttest questionnaires [32], 1 study used only focus groups [33], and 1 study used only pretest and posttest questionnaires [24]. A mixed methods approach was used by 3 studies [30,31,35], balancing quantitative data from questionnaires with qualitative data from interviews or focus groups.

Game modalities and gamification elements were diverse (eg, avatars, challenges, informative feedback, points, levels, leaderboards, storytelling), but role play and reward systems were present in all studies. Of the 7 games, 4 were Web-based [24,31,32,34], whereas the other 3 were mobile apps [30,33,35]. One study [35] also used a social networking site. Of the 7 studies, 3 described tools that were cocreated by several stakeholders including health professionals, developers, and end users [24,33,35]. All gamified digital tools were promoted and funded through health authorities for public health purposes and universities for educational purposes. Of the 7 gamified digital tools, 5 were serious games [24,31,33-35], and the other 2 were implemented as a quiz [30] and website [32].

Infectious diseases were the specific focus of 6 studies: 3 studies addressed HPV [33-35], 2 studies addressed influenza [24,32], and 1 study addressed the combination of measles, mumps, and rubella [30]. The remaining study addressed vaccination in general [31].

Tools were mostly aimed at young players: 3 tools were developed for school-aged children [24,31,33], and 2 tools were developed for university students [32,34]. For 1 of the tools developed for children [33], evaluation data were collected also from parents. The remaining 2 tools were developed for the general population [30,35].

All studies but one [27] explicitly incorporated one or more behavioral theories: self-determination theory [33], health belief model [33,34], self-concept theory [34], theory of reasoned action and planned behavior [34], game theory [24,32], social value orientation [32], nudge theory [35], and empowerment model [30].

Positive effects of gamified interventions were reported in all 7 studies across at least one of the 3 outcomes we considered (ie, behavior, cognition, and usability/acceptability). For behavior, data were available for 5 studies [24,30-32,34]; for cognition, data were available for 3 studies [30,31,35]; and for usability/acceptability, data were available for 4 studies [30,31,33,35].

Table 1 outlines the characteristics of the 7 articles included in the review presented in chronological order. See Multimedia Appendix 1 for a description of the games and outcomes.

**Table 1:** Summary of the characteristics of the 7 studies.

Author(s) (year of publication)	Country	Journal	Research design	Sample size	Sample characteristics	Type of vaccine
Bertozzi et al (2013) [24]	United States	International Journal of Gaming and Computer-Mediated Simulations	Preplay and postplay assessments through face-to-face questionnaires	12	Lower-income, school-aged children	Influenza
Böhm et al (2014) [32]	Germany	Journal of Economic Behavior & Organization	Controlled laboratory experiment (8 sessions) with postexperiment quantitative data collected through questionnaires	180 (124 women)	College-aged social science students, mean age 23.37 years (SD 4.09 years)	Influenza
Ruiz-Lopez et al (2016) [35]	Norway	Journal of Medical Internet Research Serious Games	Mixed-methods with beta testing, focus groups (2 sessions), and self-administered questionnaires	40 for the beta testing, 6 women for the first focus group, 23 (10 girls) for the second focus group	First focus group age range 40-60 years, second focus group (high school students) age range 16-18 years	HPV^a^
Cates et al (2018) [33]	United States	Games for Health Journal	Focus group (5 sessions)	16 preteens (5 girls) and 9 parents (7 women)	11-12 years old (preteens)	HPV
Darville et al (2018) [34]	United States	Simulation & Gaming	Randomized controlled trial (2x2 fully crossed between subjects) with post-experimental quantitative data collected through questionnaires (eg, Likert scales)	108	College-aged male students (18-26 years old)	HPV
Fadda et al (2017) [30]	Italy	Journal of Medical Internet Research mHealth and uHealth	Mixed methods, including a randomized controlled trial, Web-based survey (including the Mobile App Rating Scale [37]), and qualitative telephonic interviews	140 (138 women) for the survey, 60 for the telephone interviews	Parents	MMR^b^
Eley et al (2019) [31]	United Kingdom	Journal of Medical Internet Research Serious Games	Mixed methods including pre- and post-experiment questionnaires, focus groups (26 sessions), and open-ended questions in the post-experiment questionnaire	473 (123 juniors and 350 seniors) for the questionnaire, 126 for the 26 focus groups	Students 7-16 years old	Infectious diseases

^a^HPV: human papillomavirus

^b^MMR: measles, mumps, and rubella

### Description of the Evaluation Outcomes of Individual Games

#### Flu Busters!

The outcomes of this game [24] concerned behavior in terms of real action (ie, receiving vaccination). The game was deployed with 12 school-aged children in clinic waiting rooms before their appointments. A group of physician and midwives asked them to play the game and collected their feedback. Preliminary testing demonstrated that the game was reasonably successful in achieving its stated goal (ie, 10 of the 12 children were vaccinated for the flu after playing the game). Therefore, 92% of those who played the game agreed to be vaccinated compared to 42% (5/12) of a group of 12 clinic patients at the same premises who did not play the game. This demonstrates the real-world effectiveness of the game, despite the small sample size. Of the children, 27% (3/12) was able to answer how the vaccine worked after the game, while all of the children understood that germs invading the body and altering its cells were bad and that the vaccine could help. Children especially appreciated the animations of the protagonist *Vaccine Man* wrestling with the flu virus. Through feedback, the researchers also realized that the game was effective in communicating how prevalent the flu virus is in heavily populated environments.

#### I-Vax Game

Key findings of this game [32] concerned behavior. In fact, this was a game model to simulate vaccination behavior based on real-world vaccination decisions. *I-Vax* underwent a controlled laboratory experiment involving 4 sessions with 2 treatment groups each (n=96 total), 3 different sessions with 2 treatment groups each (n=84 total), and 1 session with 1 control group. Results showed that the reaction to the game varied by personality, but that the game could contribute to a better understanding of vaccination behavior and vaccine hesitancy. Those with a positive attitude were vaccinated more often and did not alter their vaccination behaviors, while those with a neutral attitude had higher switching rates (*P*<.001). Pro-socials were more likely to get vaccinated than pro-selfs (*P*<.001). Participants experiencing adverse effects after vaccination within the game were more likely to change their attitude towards vaccination (*P*<.001). Overall, participants reported an intent to change health behaviors after playing.

#### FightHPV

Outcomes of this game-based learning tool [35] reflected both cognition and usability/acceptability. Feedback was collected from 40 participants (employees of the Cancer Registry of Norway), a focus group of 6 participants aged 40-50 years (members of the Norwegian Women’s Public Health Association), and a focus group of 23 high school students aged 16-18 years. HPV knowledge and cognition before and after playing the game was evaluated in the 22 participants from the second focus group who returned a questionnaire. Gameplay data from the beta testing study were collected using Google Analytics. Concerning cognition, after playing the game, concepts about HPV vaccination were better understood, and an increase in HPV knowledge was observed (*P*=.001). As for usability/acceptability, all those who returned the questionnaire stated that FightHPV was an appealing educational tool, 69% (18/26) reported that they liked the game, and 81% (21/26) stated that the game was challenging. Google Analytics showed that the game was easy to access and use but that players stopped the game when it became too hard.

#### Land of Secret Gardens

This interactive videogame [33] presented outcomes only on usability/acceptability. Data were collected qualitatively through 3 focus groups conducted with a total sample of 16 boys and girls aged 11-12 years distributed among the focus groups as well as 2 parallel focus groups with a total sample of 9 parents distributed among the focus groups. Input on game design and perspectives on the game concept were investigated. Land of Secret Gardens was considered acceptable by both the preteens and parents. Preteens especially liked the mixture of entertaining and instructional elements and the opportunity to earn tokens and advance levels. Parents also favored game levels that were contingent on correct answers to HPV knowledge questions. However, some parents expressed hesitancy around games as motivational tools. In general, the game was appreciated as an opportunity to enhance communication about HPV between preteens and parents.

#### VAX!

This study [34] studied outcomes on behavior only. This randomized control trial involved 24-29 students per condition group. The effects of avatar characters (assigned or customized) and perception of self (ideal or actual) on HPV risk perception, HPV vaccine self-efficacy, and behavioral intent to receive the HPV vaccine were tested. Outcomes of the evaluation were perception of self, self-efficacy, and HPV vaccine intention. Although results were not statistically significant (*P* values ranging from .581 to .001), data analysis indicated an increase in gain scores for risk perception, self-efficacy, and behavioral intention when participants were able to customize their avatar to look like their ideal or actual self. After playing the game, participants declared their intent to get vaccinated.

#### Morbiquiz

The outcomes of Morbiquiz [30] concerned behavior, cognition, and usability/acceptability. The game was evaluated through a randomized controlled trial and mixed-methods with a Web-based survey (n=140) and qualitative telephonic interviews (n=60) to explore participants’ experiences with the app. Objective outcomes were measured using an adapted version of the Mobile App Rating Scale [37] corresponding to 4 objective qualities (engagement, functionality, aesthetics, and information quality) and 2 subjective qualities (star-rated question and possible recommendation of the app). In addition, 3 items were included to assess participants’ perceived impact of the app on their knowledge, on their help seeking, and the perceived likelihood of an actual change in the target health behavior. Concerning behavior, players reported significantly higher intention to vaccinate (*P*=.03) and more confidence in the decision (*P*=.006). When compared with the control group (empowerment and knowledge intervention), those receiving the app intervention were more likely to actually change their behavior and look for health information to opt for vaccination. As for cognition, all experimental groups reported a significant increase in their vaccination knowledge compared with the control group (*P*<.001). Concerning usability/acceptability, functionality and aesthetics scored high. The results of the focus group were a general appreciation of the design and content of the app. Participants defined the app as useful, trustworthy, innovative, and engaging and described their experience as fun and pleasant. Most participants reported that MorbiQuiz was highly convenient, meaning that it is handy, quick, noninvasive, easily accessible, and functional, and stressed that the game invited users to seek information actively thanks to its gamified approach.

#### Stop the Spread

The outcomes of Stop the Spread [30] concerned behavior, cognition, and usability/acceptability. A total of 123 junior-aged students and 350 senior-aged students (age range, 7-16 years) from 5 UK educational provisions completed knowledge and evaluation questionnaires before and after using the game. Focus groups with 126 students were also conducted. As for behavior, in some focus groups, students reported an intent to change their health behavior. Concerning cognition, after playing, participants reported that their knowledge about sneezing behaviors and vaccinations increased significantly (*P*<.05) for both age groups. Concerning usability/acceptability, the mean enjoyment score for Stop the Spread was 6.2/10 for juniors and 5.1/10 for seniors; participants found that Stop the Spread was fast-paced and challenging. Overall, many students reported positive perceptions of their user experience, with a few suggestions for improvement.

## Discussion

### Principal Findings

We identified 7 studies reporting data on the evaluation of the effectiveness of digital tools using gamification on the topic of vaccination. All 7 studies presented positive results in terms of pre-established outcomes (ie, behavior, cognition, and usability/acceptability) confirming that gamified digital tools can facilitate communication of vaccination-related messages and contribute to increased vaccination uptake. These results agree with those of previous studies demonstrating that gamification can contribute to changed behaviors and increased knowledge as well as be appreciated by users with regard to health-related topics [38]. When combining gamification and emerging technologies, results might be even more promising [39]. Digital tools have the advantage of being ubiquitous without time and space constraints [40], and the inclusion of gamified features might increase their appeal and acceptability, as reported in our study. Based on these assumptions, the design of future interventions should consider the use of both new technologies and gamification.

Previous research described existing serious games used for vaccination [23]. However, selected tools dated back to the year 2015 and were just listed and fully described without any appraisal of their effectiveness. Furthermore, only serious games were analyzed in this previous study [23], without considering other digital tools that were not full-blown games but used gamified features [20]. Through our review, we provide an update, through the year 2019, of existing digital games for vaccination and an evaluation of these games. Given the speed at which technology changes and improves, monitoring new digital tools is essential. Most importantly, to our knowledge, our study is the first scoping review synthesizing data on the evaluation of the effectiveness of different types of digital tools using gamification, not exclusively serious games.

We classified games per type of outcome. The 4 studies [24,30,32,34] reporting data on behavior showed a remarkable increase in the intent to get vaccinated and a positive attitude towards vaccination. Similar results on behavior change have been found in serious games for oral hygiene [41], asthma [42], and fruit and vegetable consumption [43]. Effectiveness in terms of behavior change might be explained by the fact that gamification tends to improve the involvement and motivation of users who feel more convinced of their decision after playing [44,45]. Games are thought to provide a good medium for increasing self-efficacy and changing behavior as they offer the opportunity for a new experience in a safe environment, without real-life consequences to making wrong decisions [46].

Positive findings on cognition were reported by 3 studies [30,31,35], corresponding to increased knowledge and literacy about vaccination. This agrees with a meta-analysis of serious digital games for healthy lifestyle promotion [46]. According to this previous scientific work, health knowledge is easier to influence than other outcomes, but the impact of a change in knowledge is not as strong as influencing a person’s intent to change behavior. These positive results might be explained by the learning-by-doing approach used in gamified digital tools where players learn through exploration and experimentation.

In terms of usability/acceptability, results on this outcome were reported by 3 studies [30,31,35]. Effectiveness in terms of usability is potentially justified by the co-construction process. Including different stakeholders like health professionals and game developers is time-consuming but is useful for developing games that will appeal to the target audience [47]. One game promoting healthy eating in children [48] and one game to help young people quit smoking [49] underlined the advantages of using a co-construction approach to increase commitment to and acceptability of the games.

While all 7 gamified digital tools were effective across the 3 types of outcomes, they presented different characteristics (ie, game features, targeted audience, and targeted disease for vaccination). Among successful game features, role play or characterization, earning and losing tokens, and advancing levels were the 3 modalities that were the most used and accepted by users across all studies. As for role play or characterization, FightHPV [35] and Stop the Spread [31] were appreciated by users because they included a set of characters that were considered really amusing. In particular, the avatars in Vax! [34] allowed users to better appraise risk perception for infectious diseases as well as to increase one’s intent to receive vaccination. Presenting a story with a character with which users could identify might be preferred because the character refers to one’s doubts and knowledge and helps virtually measure the impact of one’s decision. Character identification can improve risk perception and encourage vaccination uptake [50], since, within the game, the harms of nonvaccination can be virtually self-experimented. Furthermore, following a herd immunity approach [51], some of the games under study made users interact and confront with other imaginary characters to explain the collective dimension and community-level impact of vaccination. Immersive story telling could also have enhanced engagement and subsequent retention of key messages. As for earning and losing tokens, like in I-Vax [32], Land of Secret Gardens [33], and Morbiquiz [30], the presence of tokens in a game might encourage the players and maintain their motivation. The explanation can be found in behavioral theories: reinforcement and punishment contingencies are equally effective as long as they challenge the user [52]. Participants feel their role in the game is active, which makes them more engaged with the game. As for advancing levels, we know from the Stop the Spread game [31] that players enjoy the steady increase in difficulty as the game progresses. However, if games are too difficult, like the first version of FightHPV [35], users stop playing and cannot learn if they do not advance. Thus, it is important to design the game so that players feel challenged but not frustrated. Like earning and losing tokens, advancing levels maintains the interest and motivation of the player. Finally, results showed that using a serious game format, like in most of the studies [24,31,33-35], was not more effective than the other two formats (ie, quiz [30] and website [32]), thus confirming that using gamifying features might be as effective as a full-blown game. Future research including more studies is needed to validate this hypothesis.

As for the targeted audience, apart from 1 study involving parents who were a little skeptical about the trustworthiness of a gamified approach [33], all other studies targeted young people (children, students, young parents) who were attracted by digital gamified tools and represented the most captive audience. About 90% of teenagers play video games [53], and millennials are simultaneously technologically adept with and shaped by technology [54]. As future parents and adults making decisions about their health and the health of their relatives, young people represent a core target group for interventions addressing vaccination coverage.

As for the type of targeted vaccine, all games except Stop the Spread [31] targeted a specific vaccine. This might have been a good strategy to clearly explain one vaccine at a time without confusing the player. Targeting other vaccines like hepatitis, for instance, with new games might be interesting to have a complete spectrum of vaccines explained through gamified digital tools. On the other hand, the design of games seems to be independent from the type of vaccine. This means that the same game might be transferable to all types of vaccine. This could be the case of games like Morbiquiz [30], VAX! [34], and FightHPV [35], whose designs can be easily adapted to other vaccines or vaccine-related diseases.

Finally, the main findings of this review include the relevance of incorporating behavioral theories within the game conception. Almost all gamified digital tools under study were based on solid theories and proved to be effective in facilitating understanding and appraisal of information about vaccines and behavior change endpoints. As suggested by previous literature, theory-driven interventions are more efficacious than those that are not [55], which might further explain the positive outcomes of synthesized tools supported by the gamification approach.

Limitations of this review were the comparative paucity of included studies and the marked heterogeneity of their study design and contents. Publication bias should also be considered, as small studies with negative findings would likely not be published. Furthermore, the scores of some articles were low [33,34] for various reasons including a limited study focus, a research design that was not completely appropriate, nongeneralizable findings, or small sample sizes. Only 3 articles [30,31,34] presented a solid evaluation methodology relying on quantitative and qualitative pretest and posttest data. We also relied on the work of a single coder, which might have introduced systematic bias. However, by omitting a second coder, we wished to ensure consistency in the study selection. Finally, all 7 studies presented positive short-term outcomes, while no long-term impact assessment of the games was conducted.

### Recommendations for the Design of Future Interventions

Based on our study, we can suggest how to improve the design of future interventions. Especially during this period of disinformation about vaccination circulating on the internet, gamified digital tools can help provide more accurate information, while being fun and engaging. These recommendations might ensure that produced games are attractive, validated, and effective, especially if they are produced with researchers and professionals in the vaccine domain.

First, the educational perspective is fundamental for all games, but it must be implicit. Interventions should be focused on increasing knowledge and influencing behavior about vaccination without clearly presenting their final aim. Users might unconsciously learn by playing without having the feeling that they are following a training class or didactic presentation. It is important to use a narrative approach with an appropriate story line to engage, motivate, and empower users throughout the learning process. However, the information provided must be factual and trustworthy.

Second, emotional engagement between the player and the environment should be created. Fun contributes to such engagement. Using characters and avatars is a good strategy to capture and maintain the attention of the players. Avatars can self-represent the player in a simulation or role-playing game so that players are immersed in the game; if users are active, their chances to learn increase. Emotional engagement is also reinforced with levels and tokens. Their use is highly recommended for future gamified digital tools. Provide a leaderboard for competition not only with other players but also with oneself as a personal challenge. However, game functions like recovery aids and the pace of the games should be adapted to the public, to avoid frustration with levels that are too difficult.

Third, a balance should be found between simplicity and attractiveness. Future games should pay attention to the design and aesthetics of the game. Using concise text might help with retention of information and knowledge about vaccination, while not stopping the flow of the game. Amusing characters and animations are necessary to make the game appealing.

Fourth, games should take into consideration their target audience. The best solution to achieve this is to co-construct the games with all concerned stakeholders, including end users, through a design-thinking approach [56]. In general, games addressing children and young adults (especially boys) have the advantage of targeting a population who is familiar with video games and computers in general. This might not be the case for older adults.

Finally, before designing a gamified digital intervention, it is important to consider its costs, which are usually very high (at least US $10,000).

### Implications for Future Research

Our review suggests that there is a need to continue developing gamified digital tools but also to evaluate the impact of existing and future tools. Great enthusiasm for these games has led to many being produced with insufficient validation of their effectiveness. To better evaluate gamified digital tools in the short term, medium term, and long term, preferred study designs are randomized controlled trials or any other experimental design with a control group, combined with longitudinal data collection. Using a mixed methods design would also be beneficial to comprehensively capture users’ opinions and satisfaction with the game. Like in our scoping review, the outcomes to measure might be behavioral indicators like individual vaccination records or intent to get vaccinated, cognitive indicators like an increase in knowledge or vaccine literacy, and indicators of usability/acceptability of the intervention. If possible, all 3 types of outcomes should be measured to better assess the overall qualities of the games. Instruments like the Mobile App Rating Scale [37] could be used for this purpose. These evaluation studies will eventually help us understand how gamified digital tools can change vaccination uptake and coverage.

### Conclusions

Gamification is an innovative and promising option to consider when designing vaccination-related interventions addressed to the general public and young people in particular, especially for those who are hesitant about vaccination. Based on the findings of this review, health professionals, health promotion and prevention specialists, and developers are encouraged to use game-based features in interventions aimed to endorse vaccination uptake in order to increase their acceptability and consequent effectiveness. Theory-driven gamified digital tools are preferred.

## Appendix

Multimedia Appendix 1 Summary of the findings on gamified digital tools in the 7 studies.

## Abbreviations

HPV: human papillomavirus.
MMR: measles, mumps, and rubella.
