# Supplementary material for: Digital Gamification to Enhance Vaccine Knowledge and Uptake: Scoping Review
Source: JMIR Serious Games. 2020 May 18;8(2):e16983. doi: 10.2196/16983 (PMC7265110; doi:10.2196/16983)
Supplement: Multimedia Appendix 1 [file games_v8i2e16983_app1.docx]

**Multimedia Appendix 1**

**Summary of the findings on gamified digital tools in the 7 studies.**

| Author(s) | Theory | Game content | Game modality | Gamification element(s) | Data analysis | Type of outcome | Mean quality score |
| --- | --- | --- | --- | --- | --- | --- | --- |
| Bertozzi et al [24] | Game theory | In “Flu Busters,” Vaccine Man helps keep the player safe in a school environment full of sneezing, coughing, and otherwise virus distributing agents. Players help him accomplish this mission. | Web-based game, role play | Serious game | Descriptive analyses | Behavior | 6 |
| Böhm et al [32] | Game theory, social value orientation | “I-Vax” is a gamified experiment with 2 separate parts: an individual decision-making task and a group decision-making task. Each player is endowed with ECUs^a^ in each round and has to choose exactly one behavioral action representing non-vaccination and vaccination, respectively. Players earn points for good decisions, which are converted into money. | Computerized decision-making experiment, levels, points (currency units) | Leaderboard | Regression models | Behavior | 7 |
| Ruiz-Lopez et al [35] | Nudge theory | In “FightHPV,” the characters Epithelial cell, Low-risk HPV^b^, High-risk HPV, Wart, Ointment, Precancerous cells, Excision, Intercourse, Prevention Method, HPV Vaccine, Immune System, HPV Antibody, Exfoliated cell, and Screening are displayed on a frame of game board–based puzzles. The main character is Epithelial cell, and the challenge is to unify all the Epithelial cells on the game board by changing their positions swiftly using as few moves as possible for a total of 60 levels. | Game-based mobile app (iOS or Android), puzzles, levels, points, leaderboard, social networking | Serious game | Descriptive analysis for beta testing, thematic analysis for the focus groups, and descriptive analyses for the questionnaires | Cognition; usability/acceptability | 13 |
| Cates et al [33] | Self-determination theory; health belief model | The first scene of the “Land of Secret Gardens” shows planting beds without plants. Through interactive “mini-games,” players are pulled into the game by teaching them to protect their bodies -or “secret gardens” - from HPV through vaccination. Coins are earned for completing the mini games, allowing the preteen to purchase supplies for planting seeds in their garden. As more coins are earned, players can create a potion or vaccine to protect their plants from diseases or HPV. Successful protection ultimately results in mature plants being covered by shields. | Single-player videogame on tablets, smartphones and personal computers (IOS or Android); points, rewards, levels, challenges, and leaderboard | Serious game | Thematic analysis by constant comparison (grounded theory technique) | Usability/acceptability | 8 |
| Darville et al [34] | Health belief model, self-concept theory, theory of reasoned action and planned behavior | In “VAX!,” players are tasked with preparing for an outbreak by vaccinating a network that resembles human social networks. After distributing vaccines, an infectious outbreak begins to spread, and the player is tasked with quelling the epidemic by quarantining individuals at risk of becoming infected. | Interactive videogame, avatar customization, role playing, puzzles | Serious game | Factorial ANOVA with repeated measures, split plot | Behavior | 9 |
| Fadda et al [30] | Empowerment model | “Morbiquiz” is a quiz that asks players every day for 10 days to answer a series of short questions about MMR^c^ vaccination. By answering the questions, players receive content that provides information and news about this vaccination. If players answer the questions exactly, they can collect points and challenge other users who take part in the quiz. | Game-based mobile app, quiz (iOS or Android), points, levels | Leaderboard | Descriptive statistics and regression models for quantitative data, thematic analysis for qualitative data | Behavior,  cognition,  usability/acceptability | 14 |
| Eley et al [31] | N/A | In “Stop the Spread,” players prevent the spread of infection in a school by catching sneezes with a tissue and throwing the used tissue into a bin. As sneezes go uncaught, more children in the playground become infected. The game is over once every child in the playground is infected. The longer the game lasts, the more points the player scores. An additional level includes the concept of vaccination and herd immunity. In this level, players vaccinate children in the playground as quickly as they can to slow the spread of infection. | Interactive videogame, levels, points | Serious game | Descriptive statistics and regression models for quantitative data, thematic analysis for qualitative data | Behavior, cognition, usability/acceptability | 14 |
